# Supplementary material for: In vivo pharmacokinetics of ginsenoside compound K mediated by gut microbiota
Source: PLoS One. 2024 Aug 23;19(8):e0307286. doi: 10.1371/journal.pone.0307286 (PMC11343376; doi:10.1371/journal.pone.0307286)
Supplement: Attachment — Submitted filename: letter for response.doc [file pone.0307286.s001.doc]

***PLOS ONE***

26 Apr 2024

# Responses to Editor’s and Reviewers’ Comments

Dear Editor-in-chief,

We are submitting a revised manuscript entitled “**In vivo pharmacokinetics of ginsenoside compound K mediated by gut microbiota**” by Deng *et al*. for publication as a full paper in the ***PLOS ONE*.** We have carefully considered your comments in preparing the revised manuscript. We would like to give a point-by-point response to the concerns. The main manuscript has been revised accordingly. Here, we have enclosed the replies to the reviewers’ comments,

***Reviewer’s comments***,

*1. In the section ‘Instruments and condition’ MRM transitions of drug molecules were not mentioned. No quantification can be done without this information.*

**R:** Thanks for your comments. The MRM transitions of drug molecules were supplemented in Figure 2.

*2. Collision gas and its flow rate not mentioned.*

**R:** Thanks for your comments. The flow rate is 0.3 mL/min; collision gas is 9. We have supplemented this information in the revised manuscript.

*3. No* *re-equilibration time has been provided to the system which is essential for a gradient elution program.*

**R:** Thanks for your comments. The re-equilibration time was set as 5 min in this study. We have supplemented it in the revised manuscript.

*4. Calibration standards and quality control: analytical range not mentioned for QCs and calibration standards.*

**R:** Thanks for your comments. Calibration standards and QC were validated according to the 2018 FDA Guidelines.

5. *System suitability parameters (tailing factor, theoretical plates) have not been mentioned.*

**R:** Thanks for your comments. The tailing factors under the selected chromatographic conditions were calculated as 0.993 and 0.996 for GCK and PPD, respectively, while the theoretical plates were measured as 1.13 × 10^4^. We have calculated the chromatographic parameters, which have been added in the manuscript.

6. *The chromatograms are not legible. No information about Molecular ion peaks or daughter ions has been provided.*

**R:** Thanks for your comments, we have re-provide clearer chromatograms.

7. *English usage is not up to the standard. Phrases like ‘all animals were intra-gastric administration’ and ‘The peak areas near all the analytes in the blank plasma were satisfied less than 20% of all the analytes’ are few examples.*

**R:** Thanks for your comments, we have corrected and polished the language.

I hope you will find our corrections worthy of publication in the ***PLOS ONE***.

Best regards.

Yours Sincerely,

Zheng-Guang Wang, PhD

Department of Spinal Surgery,

The third Xiangya Hospital, Central South University
